# Spatiotemporal composition and diversity of endophyte communities in *Dracaena cambodiana* on Hainan Island

**DOI:** 10.3389/fmicb.2025.1540669

**Published:** 2025-02-28

**Authors:** Sipeng Li, Yang Liu, Xin Yang, Yun Yang, Junxiang Peng, Yanhong Xu, Jianhe Wei

**Affiliations:** ^1^Hainan Provincial Key Laboratory of Resources Conservation and Development of Southern Medicine and Key Laboratory of State Administration of Traditional Chinese Medicine for Agarwood Sustainable Utilization, Hainan Branch of the Institute of Medicinal Plant Development, Chinese Academy of Medical Sciences and Peking Union Medical College, Haikou, China; ^2^Key Laboratory of Bioactive Substances and Resources Utilization of Chinese Herbal Medicine, Ministry of Education and National Engineering Laboratory for Breeding of Endangered Medicinal Materials, Institute of Medicinal Plant Development, Chinese Academy of Medical Sciences and Peking Union Medical College, Beijing, China

**Keywords:** *Dracaena cambodiana*, endophytic fungi, secondary metabolite biosynthesis, signal transduction, metagenomic sequencing

## Abstract

**Introduction:**

*Dracaena cambodiana* produces a red resin known as Dragon’s blood, which is used worldwide in traditional medicine and as a dye. The role of endophytes in the resin-formation process remains underexplored. Understanding the endophyte communities and their functional roles in resin production could enable the development of efficient induction techniques for resin production.

**Methods:**

In this study, ITS and metagenomic sequencing analyzed endophyte communities’ characteristics and functional traits in different tissues and *D. cambodiana* across multiple wild populations on Hainan Island.

**Results:**

We identified distinct fungal genera that were dominant in different tissues. Following injury, we observed significant changes in the expression of endophytic fungal genes. These changes indicated that metabolic pathways associated with resin metabolism, sucrose metabolism, signal transduction, and phenylalanine metabolism were likely involved in resin formation. Additionally, several glycosylation gene families were upregulated in the post-injury endophytic communities, which suggests a role in flavonoid transport and the reduction of autotoxic effects.

**Discussion:**

Our results suggest that endophytes play a vital role in the resin-formation process of *D. cambodiana*. Isolating specific endophytes or using synthetic communities could potentially improve resin yields and avoid pathogenic fungi, ensuring safety. The findings from this study provide a theoretical basis for the development of high-efficiency resin induction techniques by targeting the dynamic changes in endophyte communities across tissues, regions, and resin formation stages.

## 1 Introduction

Dragon trees represent a distinct category of *Dracaena* spp. (Asparagaceae) characterized by their aesthetically pleasing, tree—like morphology. These plants are among the oldest known, first emerging during the Cretaceous period (Aslam et al., 2013). The name dragon tree refers to the ability of this species to excrete red resin, known as dragon’s blood, after the trees are damaged (Zheng et al., 2009; Ibraheam et al., 2018). In the past of many civilizations, dragon ‘s blood has been a precious traditional medicine and valuable crimson coating material (Wang et al., 2011). Along with three subspecies of the *Dracaena* genus, the dragon trees comprised 11 recognized species (Benabid and Cuzin, 1997; Marrero et al., 1998; Marrero and Almeida Pérez, 2012; Wilkin et al., 2013). The Red List of Threatened Species of the International Union for Conversation of Nature^1^ lists these species as protected while at risk. The scarcity of dragon tree resources worldwide has greatly limited the development and application of dragon’s blood.

The formation of dragon’s blood is a defensive response of dragon trees to external stress, such as mechanical injury, insect attacks, and microbial invasion (Wang et al., 2010; Ding et al., 2020). Under natural conditions, resin formation in dragon trees requires several years, resulting in extremely low yields (Wang et al., 2011), making it challenging to meet financial requirements. Therefore, methods for rapidly inducing resin need to be developed. Several studies have shown that resin formation can be induced through fungal inoculation. For instance, the accumulation of dragon’s blood in *Dracaena* spp. is considerably impacted by fungal inoculation with *Fusarium* and *Septoria* (Jura-Morawiec and Tulik, 2016). Another study investigated the enhancement of dragon’s blood production by inoculation with *Fusarium moniliforme* strains in *D. cochinchinensis* (Jiang et al., 2003), while another study focused on the use of *Fusarium oxysporum* strains (Wang et al., 2010), and yet another examined the effect of *Fusarium proliferatum* strains for the same purpose(Wang et al., 2011). These studies indicated a highly efficient induction technique based on endophytic fungi could be developed. However, owing to a lack of comprehensive understanding of the overall profile of endophytic fungi in dragon trees, the efficiency of these methods is limited and has not been applied to practical production.

*Dracaena cambodiana* is a representative dragon tree in Asia primarily distributed on Hainan Island in China (Liu et al., 2013). Through the promotion of standardized cultivation techniques, *D. cambodiana* has emerged as a prevalent ornamental tree in high-latitude regions of China, establishing itself as the most abundant and promising source of dragon’s blood globally. Although *D. cambodiana* is regarded as a standard source of dragon’s blood in many areas in China (Zhejiang, Hainan), many cultivated *D. cambodiana* have not demonstrated their expected medicinal value because they lack efficient artificial induction techniques. This study aims to elucidate the composition and diversity of the endophytic microbial communities in different tissues and habitats of *D. cambodiana* in Hainan Island, and to infer the role of endophytes in resin formation and accumulation by examining the enrichment of functional genes in the microbial community under wound stress.

## 2 Study methodology

### 2.1 Sample collection and pretreatment

In total, 50 stem samples (five samples from each population) and resin-containing stem samples (five samples from each population) were collected from 10 wild populations of *D. cambodiana* on Hainan Island. DNA was extracted from the endophytic fungi from various regions of Hainan Province for ITS and metagenomic sequencing. The samples were cut into 3 cm × 3 cm × 3 cm pieces and stored in liquid nitrogen at −80°C until that time.

Root, stem, leaf, and fruit samples (five samples from each part) of *D. cambodiana* were collected in Haikou, Hainan Province. After being surface-sterilized with 75% ethanol and washed with sterile water, all samples were kept at −80°C until DNA extraction. Internal transcribed spacer (ITS) sequencing of endophytic fungi was performed from different tissues of *D. cambodiana*.

Healthy, short-term postinjury samples and long-term postinjury samples of *D. cambodiana* were collected in Haikou. The injury was made directly on the stems as we described in our previous study (Zhang et al., 2024).Three replicates per treatment were used for metagenomic sequencing of endophytic fungi. Before DNA extraction, the samples underwent rinsing with sterile water and were surface-sterilized using 75% ethanol. Before being extracted for DNA, the samples were kept at −80°C.

### 2.2 DNA extraction, PCR, and sequencing

#### 2.2.1 DNA extraction and processing for ITS sequencing

According to the manufacturer’s instructions, microbial DNA was extracted from *D. cambodiana* stem samples using the DNeasy® PowerSoil® Kit (MOBIO Laboratories, USA). The DNA purity and concentration were then assessed via agarose gel electrophoresis. A suitable amount of sample DNA was added to a centrifuge tube, and amplification of the fungal ITS1 region was performed using the primers ITS1F (5′-CTTGGTCATTTAGAGGAAGTAA-3′) and ITS4 (5′-GCTGCGTTCTTCATCGATGC-3′). The PCR mixture consisted of 25.0 μl of 2× Taq Master Mix (Novoprotein), 2.0 μl of forward primer (10 μM), 2.0 μl of reverse primer (10 μM), 5 μl of template DNA (40–60 ng), and ddH2O, making a final volume of 50 μl. The amplification conditions were as follows: an initial denaturation at 95°C for 3 min, followed by 35 cycles of denaturation at 95°C for 20 s, annealing at 56°C for 20 s, and extension at 72°C for 30 s. A final extension was performed at 72°C for 5 min. The PCR products were then extracted from 1.5% agarose gels and purified using the E.Z.N.A.® Gel Extraction Kit (Omega). Library preparation followed the standard protocol of the NEBNext® Ultra™ DNA Library Prep Kit for Illumina®. The libraries were sequenced using the Illumina platform with paired-end sequencing.

#### 2.2.2 DNA extraction and processing for metagenomic sequencing

According to the manufacturer’s instructions, microbial DNA was extracted from *D. cambodiana* stem samples using the DNeasy® PowerSoil® Kit (MOBIO Laboratories, USA). Short segments of genomic DNA were randomly sheared. Illumina adapters were used to ligate the recovered, end-repaired, and A-tailed fragments. PCR amplification, size selection, and purification were performed to amplify the fragments, including adapters. Real-time PCR was employed for quantification, and the Qubit fluorometer was used to validate the library, while a bioanalyzer assessed the size distribution. The measured libraries were combined and sequenced on Illumina platforms, following the required data quality and effective library concentration standards. After sequencing, plant-derived sequences were removed by comparing the reads to the *D. cambodiana* genome data to eliminate host plant DNA contamination. This process ensured that only fungal sequences were included in the subsequent analysis. The clean data were then used for further processing and functional annotation.

### 2.3 Data filtering and analysis

#### 2.3.1 Filtering and analysis of ITS sequencing data

Raw tag data were obtained using FLASH to construct the reads for each sample after the primer and barcode sequences were removed (Magoč and Salzberg, 2011). The assembled raw tags underwent rigorous filtering (Bokulich et al., 2013) to yield high-quality data (clean tags). The subsequent steps were implemented following the Qiime tag quality control process. When the number of successive low-quality values (default quality threshold <= 19) surpassed the predetermined length (default length value = 3), raw tags were terminated at the first low-quality base site. Tag length filtering involved the removal of tags from the dataset that exhibited a consecutive high-quality base length of less than 75% of the total tag length after truncation. The processed tags were examined for the presence of chimeric sequences. The final effective tags were obtained by comparing tag sequences to the species annotation database (Rognes et al., 2016) to identify chimeric sequences. These sequences were then eliminated (Haas et al., 2011).

The UPARSE software (Edgar, 2013) was used to cluster all effective tags from all samples. Sequences were grouped into operational taxonomic units (OTUs) at a default identity threshold of 97%. The UPARSE technique, which identifies the most prevalent sequence inside an OTU as the representative sequence, was used to choose representative sequences for each OTU. The BLAST method was used to annotate OTU sequences for species in the Unit (v.8.2) database (Altschup et al., 1990) and the QIIME program (v.1.9.1; Kõljalg et al., 2013); the community composition of each sample was statistically analyzed. The evolutionary connections of all representative OTU sequences were ascertained by quick multiple sequence alignments using the MUSCLE (Edgar, 2004) software (v.3.8.31). The data for each sample were then standardized to the smallest sample size. The normalized data served as the basis for further alpha and beta diversity investigations. With accession number PRJNA1185629 and PRJNA1187799, the ITS sequencing findings are available in the NCBI-SRA (Sequence Read Archive).

#### 2.3.2 Filtering and analysis of metagenomic sequencing data

Readfq (V8, https://github.com/cjfields/readfq) was used to preprocess the raw data from the Illumina HiSeq sequencing platform to produce clean data for further analysis. The processing steps are outlined as follows: Reads with low-quality bases, defined by a default quality threshold value of 38 and a minimum length of 40 bp, were excluded. Furthermore, reads containing N bases that exceeded a specified percentage over a default length of 10 bp were also removed. Lastly, reads overlapping with the adapter beyond a certain threshold, with a default length of 15 bp, were eliminated. Finally, the reads that shared the overlap above a certain portion with the adapter (default length of 15 bp) were removed. Considering that host contamination may occur in samples, clean data were blasted to the host database using the Bowtie2.2.4 software (Bowtie2.2.4, http://bowtie-bio.sourceforge.net/Bowtie2/index.shtml) to filter the reads that were of host origin, and the parameters (Qin et al., 2010; Karlsson et al., 2012) used were as follows: end-to-end, sensitive, -I 200, and -X 400 (Qin et al., 2010; Karlsson et al., 2012).

MEGAHIT software (v1.0.4-beta) was employed for data assembly, using the following parameters: Preset meta-large (--min-count 2 --k-min 27 --k-max 87 --k-step 10); subsequently, the assembled scaftigs with N connections were interrupted, while the scaftigs without N were retained (Karlsson et al., 2013; Nielsen et al., 2014; Feng et al., 2015). Bowtie 2.2.4 software was used to compare the clean data of all samples to each scaffold to obtain the PE reads that were not used. The following parameters were employed (Feng et al., 2015): -sensitive, -end-to-end, -I 200, and -X 400.

The unigenes were blasted to the functional database using the DIAMOND software (V0.9.9), with the BLASTP parameter set to -e 1e-5 (Scher et al., 2013; Oh et al., 2014). The functional database excluded the KEGG (Kanehisa et al., 2006; Kanehisa et al., 2014) database (v.2018–01-01, http://www.kegg.jp/kegg/), eggNOG (Powell et al., 2014) database (v. 4.5, http://eggnogdb.embl.de/#/app/home), and CAZy (Cantarel et al., 2009) database (v.201801, http://www.cazy.org/). For the BLAST result of each sequence, the best BLAST hit was used for subsequent analysis (Scher et al., 2013; Oh et al., 2014; Bäckhed et al., 2015). The relative abundance of various functional hierarchies is defined as the sum of the relative abundance assigned to each functional level. The function annotation findings and gene abundance table were used to generate the gene number table of each sample in each taxonomic hierarchy, where the gene number of a function in a sample is equal to the gene number annotated to that function, and the abundance is nonzero. We counted annotated gene numbers, evaluated the overall relative abundance situation, developed an abundance cluster heat map, conducted a decrease-dimension analysis of PCA and NMDS, and carried out ANOSIM of the differences between groups (inside) based on the functional abundance, metabolic pathway comparison, and Metatat and LEfSe analysis of functional differences between groups. All of these procedures were based on the abundance table of each taxonomic hierarchy. The NCBI-SRA (Sequence Read Archive) includes the metagenomic analysis results under accession numbers PRJNA1185199 and PRJNA1185465.

#### 2.3.3 Metabolite extraction and detection

Stem samples are freeze-dried by vacuum freeze-dryer (Scientz-100F). The freeze-dried sample was crushed using a mixer mill (MM 400, Retsch) with a zirconia bead for 1.5 min at 30 Hz. Dissolve 100 mg of lyophilized powder with 1.2 ml 70% methanol solution for 6 times in total. The sample extracts were analyzed using an UPLC-ESI-MS/MS system (UPLC, SHIMADZU Nexera X2, https://www.shimadzu.com.cn/; MS, Applied Biosystems 4500 Q TRAP, https://www.thermofisher.cn/cn/zh/home/brands/applied-biosystems.html). The analytical conditions were described in previous study (Liu et al., 2022).

#### 2.3.4 Data analysis and visualization

Personalized statistics, differential analysis and visualization application based on sequencing and chemical raw data were conducted using the Omicshare platform (Mu et al., 2024).

## 3 Results

### 3.1 Composition and diversity of endophytic fungal communities in different tissues of *D. cambodiana*

Endophytes in the roots, stems, leaves, and fruits of *D. cambodiana* were analyzed. The stems were divided into three parts based on their anatomical characteristics: the bark, primary, and secondary growth regions. The ITS1 region of the endophytic fungi from each tissue was sequenced. In total, 4,076,075 reads were detected. After low-quality chimeric sequences were filtered out, 3,133,276 high-quality sequences were obtained. In total, 19,273 OTUs were produced after these sequences were grouped into OTUs at 97% similarity.

**Figure 1:**
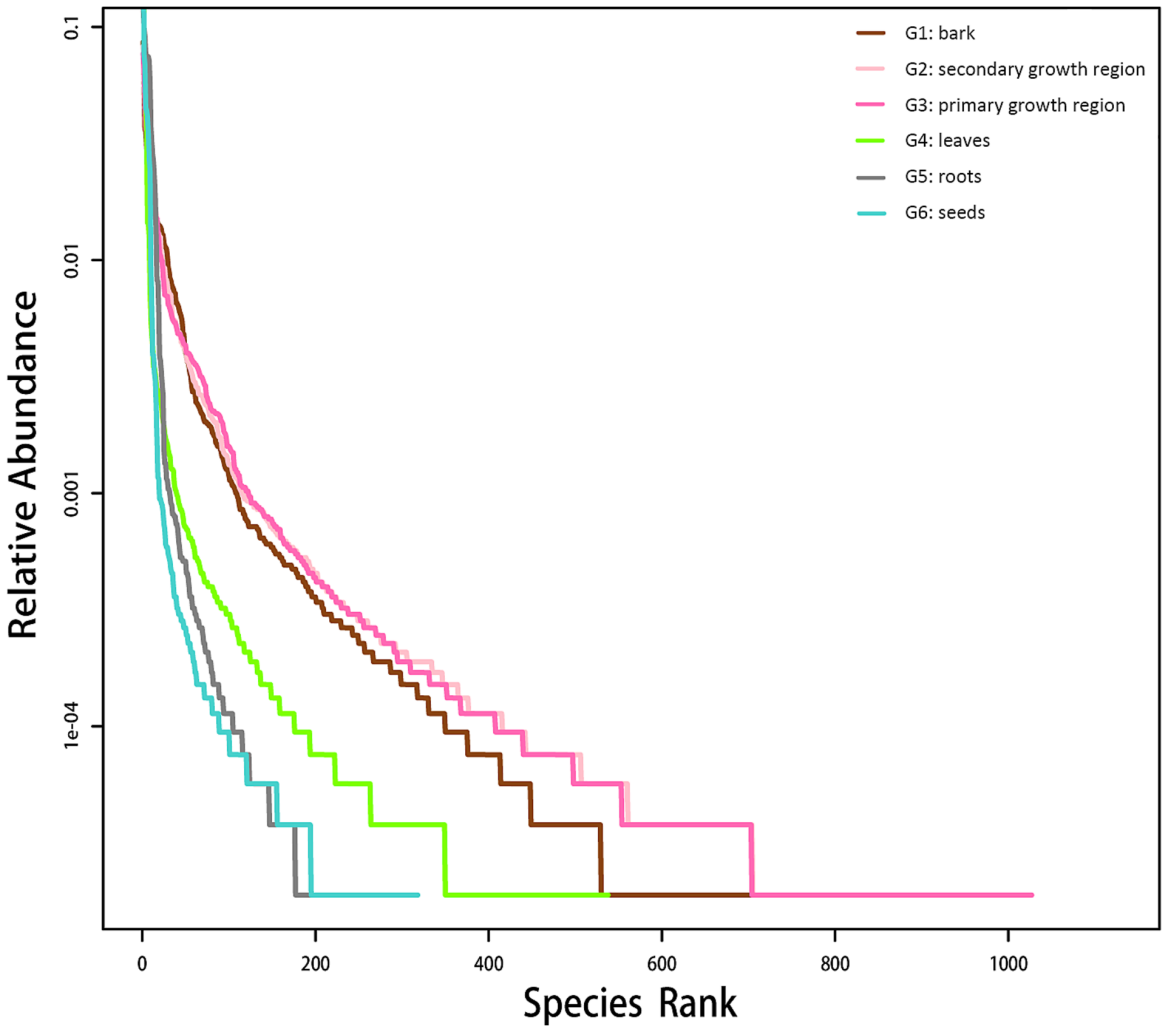
Endophytic fungal rank-abundance curve in various *D. cambodiana* tissues. G1: bark, G2: secondary growth region, G3: primary growth region, G4: leaves, G5: roots, and G6: seeds.

The rank-abundance curve was used to interpret species richness and evenness. As shown in Figure 1, the curve gradually leveled off as the sample size increased, which indicated that additional sampling did not significantly increase the number of new OTUs. These findings suggested that the sequencing depth was sufficient to capture the species diversity and richness within the samples.

**Figure 2:**
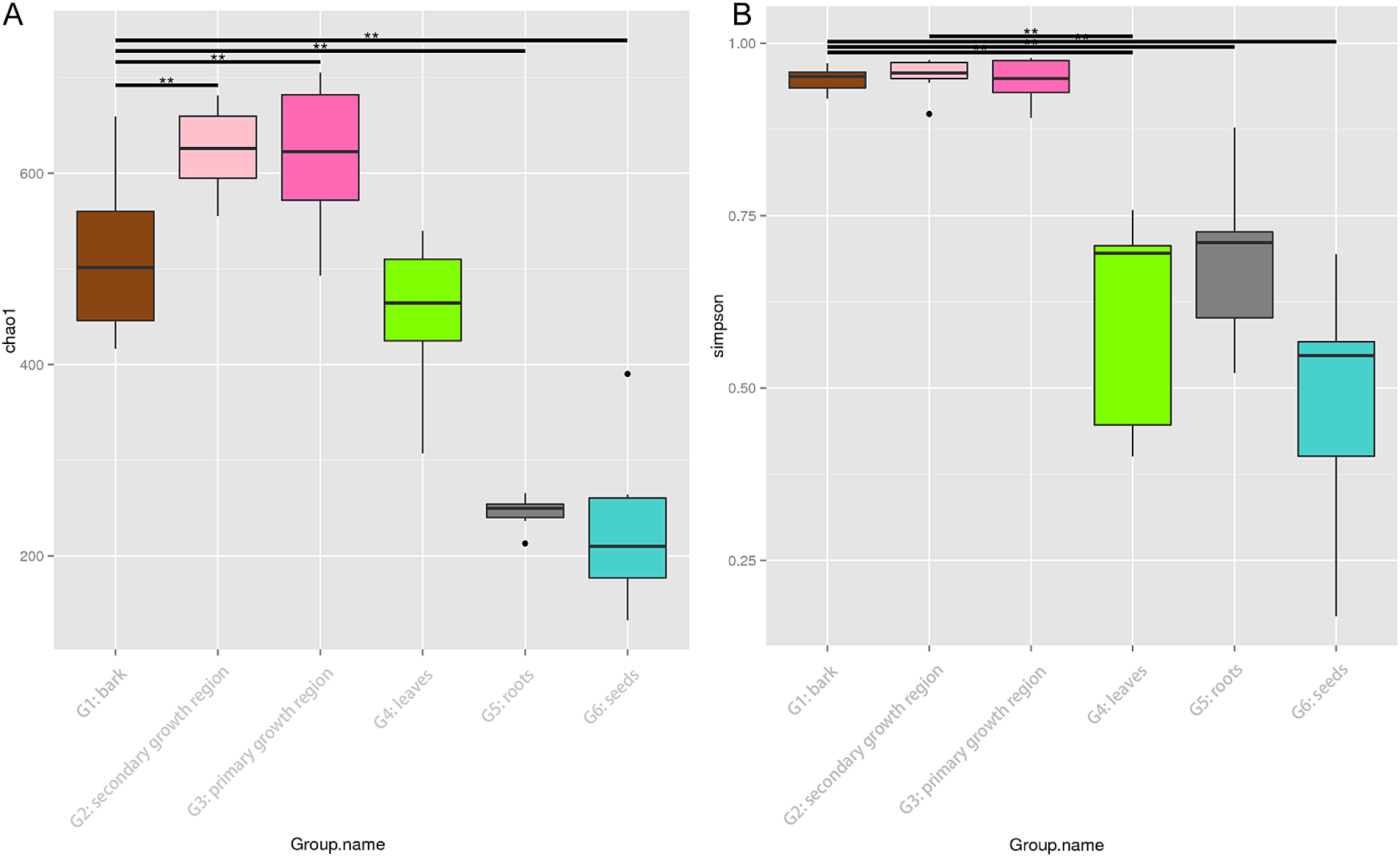
Chao1 and Simpson diversity indices of endophytic fungi in different tissues of *D. cambodiana*. **(A)** Boxplot of the Chao1 diversity index. **(B)** Boxplot of the Simpson diversity index. Significant differences between groups are indicated by * and ** (*p* < 0.05 and *p* < 0.01, respectively). G1: bark, G2: secondary growth region, G3: primary growth region, G4: leaves, G5: roots, and G6: seeds.

Figure 2 shows boxplots of the Chao1 and Simpson indices for different sample groups, which were used to estimate species richness and diversity. The boxplot of the Chao1 index (Figure 2A) revealed that the endophytes in the secondary growth region presented the highest species richness. On the other hand, the G6 group demonstrated the lowest species richness, with notable variability among the groups (*p* < 0.01). The boxplot of the Simpson index (Figure 2B) indicated that the G1, G2, and G3 groups showed higher species diversity, while the G6 group demonstrated the lowest diversity, with substantial intergroup differences (*p* < 0.01). No substantial differences were observed across the G1, G2, and G3 groups; however, these groups showed substantial variations compared to the G4, G5, and G6 groups.

The results demonstrated notable disparities in the alpha diversity indices among the groups, with intergroup variation exceeding intragroup variation. The G2 group presented the highest species richness and diversity, whereas the G6 group presented the lowest species richness and diversity. The results of this study confirmed the substantial disparities in endophytic fungal communities across various tissue types.

**Figure 3:**
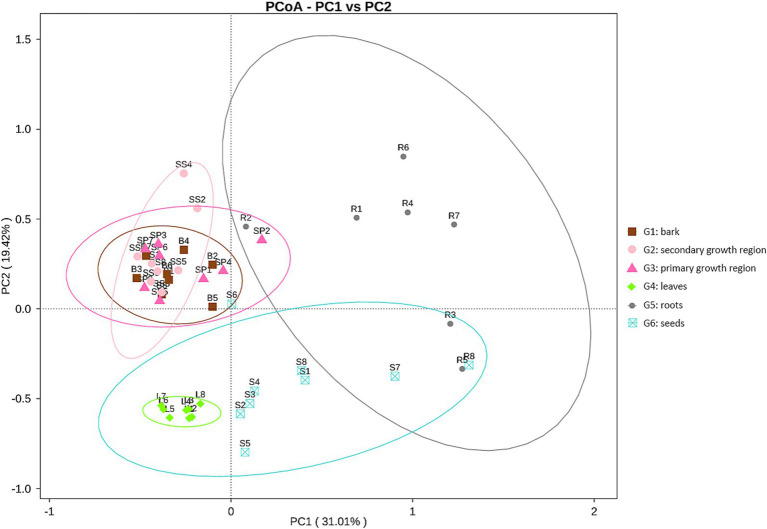
PCoA plot of endophytic fungal communities in different tissues of *D. cambodiana*. G1: bark, G2: secondary growth region, G3: primary growth region, G4: leaves, G5: roots, G6: seeds.

The Bray-Curtis principal coordinate analysis (PCoA; Figure 3) demonstrates the variations in microbial community structure across distinct sample groups. The plot illustrated the distribution of each sample group over two primary coordinate axes, with PCoA1 and PCoA2 accounting for 31.01 and 19.42% of the community variation, respectively. The compositions of the endophytic microbial communities in the roots, stems, leaves, and seeds differed significantly. The greater complexity of the microbial environment in the soil led to greater intragroup variation in the root endophytic community composition. In contrast, the endophytic community in leaves was more stable, while the compositions of the endophytic communities in the three parts of stem tissue were more similar.

**Figure 4:**
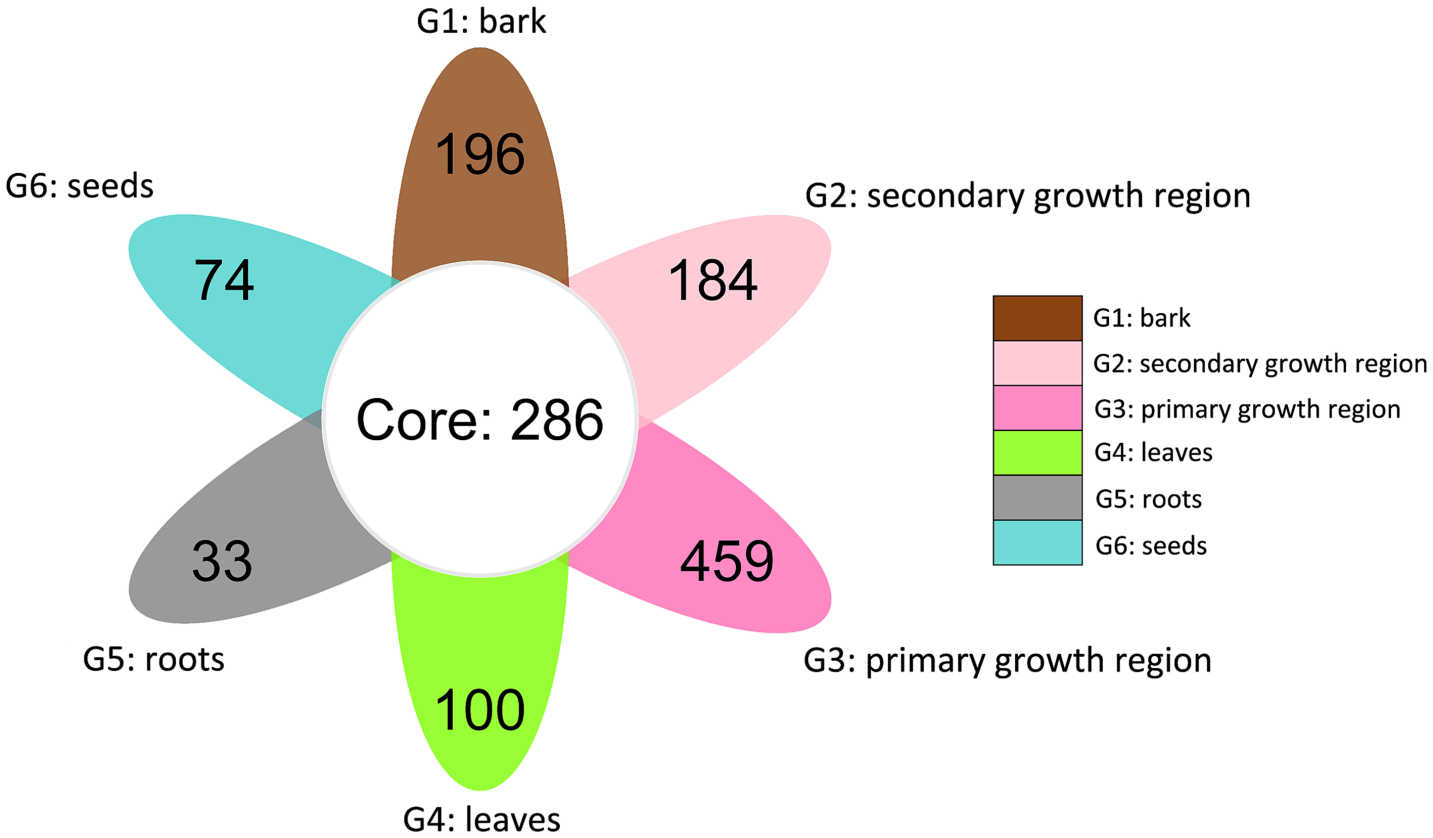
Venn diagram of shared and distinct endophytic fungal OTUs across different tissues of *D. cambodiana*. G1: bark, G2: secondary growth region, G3: primary growth region, G4: leaves, G5: roots, and G6: seeds.

The petal diagram (Figure 4) showed that the unique OTU counts for groups G1, G2, G3, G4, G5, and G6 were 196, 184, 459, 100, 33, and 74, respectively, with 286 core OTUs shared across all groups. These results indicated significant diversity within the fungal communities of each structure, along with a portion of shared core communities.

**Figure 5:**
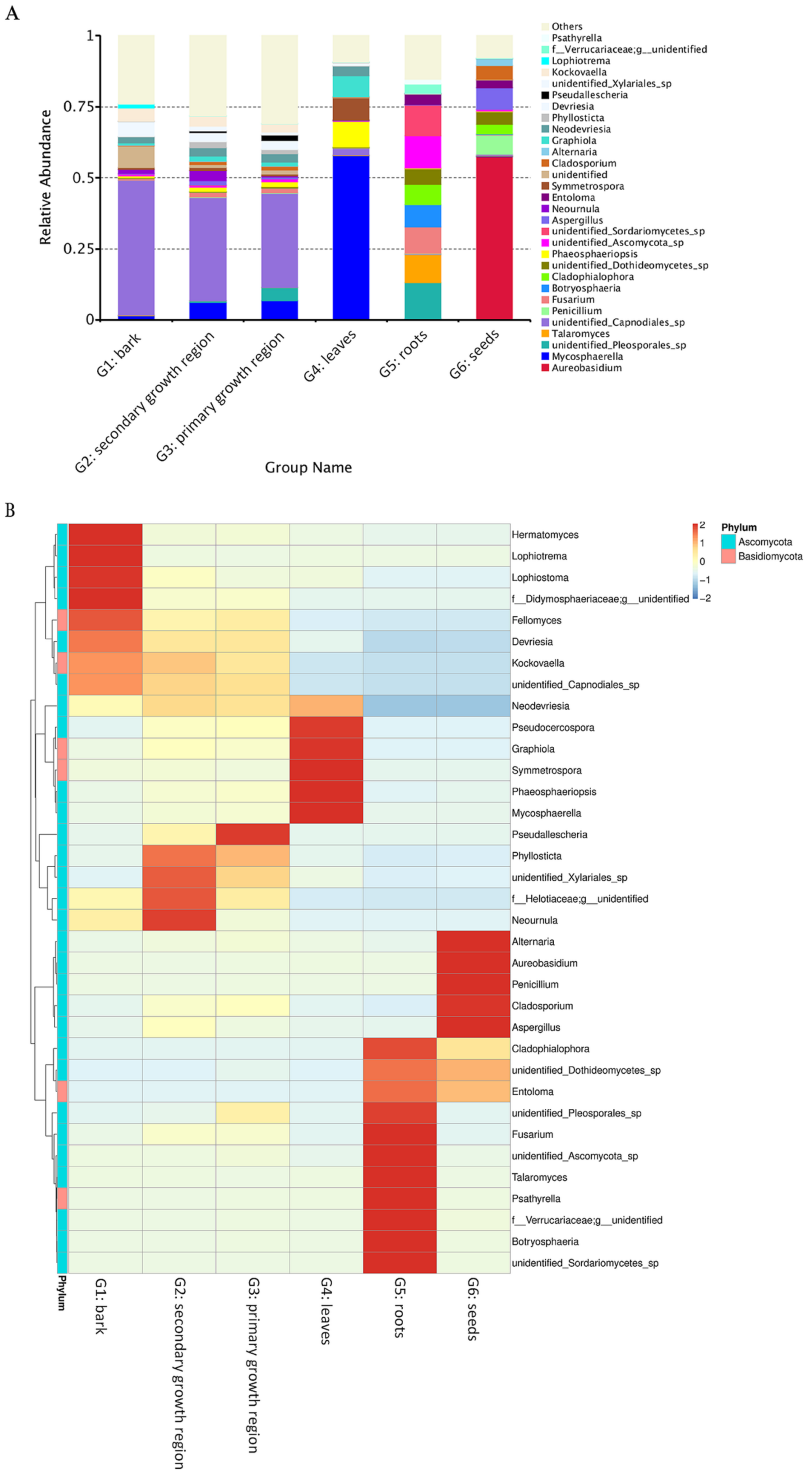
Heat map and relative abundance of endophytic fungal populations in several *D. cambodiana* tissues. **(A)** Stacked bar plot of fungal genus-level relative abundance across various tissue groups of *D. cambodiana*. **(B)** Heat map of fungal genus abundance in different tissue types. G1: bark, G2: secondary growth region, G3: primary growth region, G4: leaves, G5: roots, G6: seeds.

The relative abundance analysis showed that Ascomycota was the most prevalent fungal phylum across all groups (Figure 5A), with a relative abundance ranging from 72.20 to 91.43%. Dothideomycetes was the primary class within Ascomycota, with Pleosporales and Capnodiales as the major orders across groups. In contrast, Dothideales was the leading order in G6, accounting for 57.41% of the relative abundance of fungi (Figure 5A). At the genus level, G1, G2, and G3 had partially overlapping dominant genera, but each had unique dominant genera (Figure 5B). For example, all three groups presented high abundances of *Devriesia* and *Kockovaella*. However, *Lophiotrema* (1.41%) and *Lophiostoma* (1.39%) were the unique dominant genera in G1, *Neournula* (3.68%) in G2, and *Pseudallescheria* (1.80%) in G3. In contrast, the dominant genera in G4, G5, and G6 were different from each other and significantly different from those in G1, G2, and G3. In G4, the unique dominant genera were *Graphiola* (7.27%), *Symmetrospora* (7.82%), *Phaeosphaeriopsis* (8.96%), and *Mycosphaerella* (57.55%). In G5, *Fusarium* (9.38%), *Talaromyces* (9.91%), and *Psathyrella* (1.54%) were dominant, whereas in G6, *Alternaria* (2.38%), *Aureobasidium* (57.40%), *Penicillium* (6.94%), and *Cladosporium* (4.90%) were predominant (Figure 5). These findings indicated that in *D. cambodiana*, certain fungal species exhibit significant tissue-specific parasitism. These dominant fungi in the endophytic communities of specific tissues may influence the growth, development, and physiological functions of different tissues in *D. cambodiana*.

### 3.2 Composition and diversity of endophytic fungal communities in *D. cambodiana* from different regions

**Figure 6:**
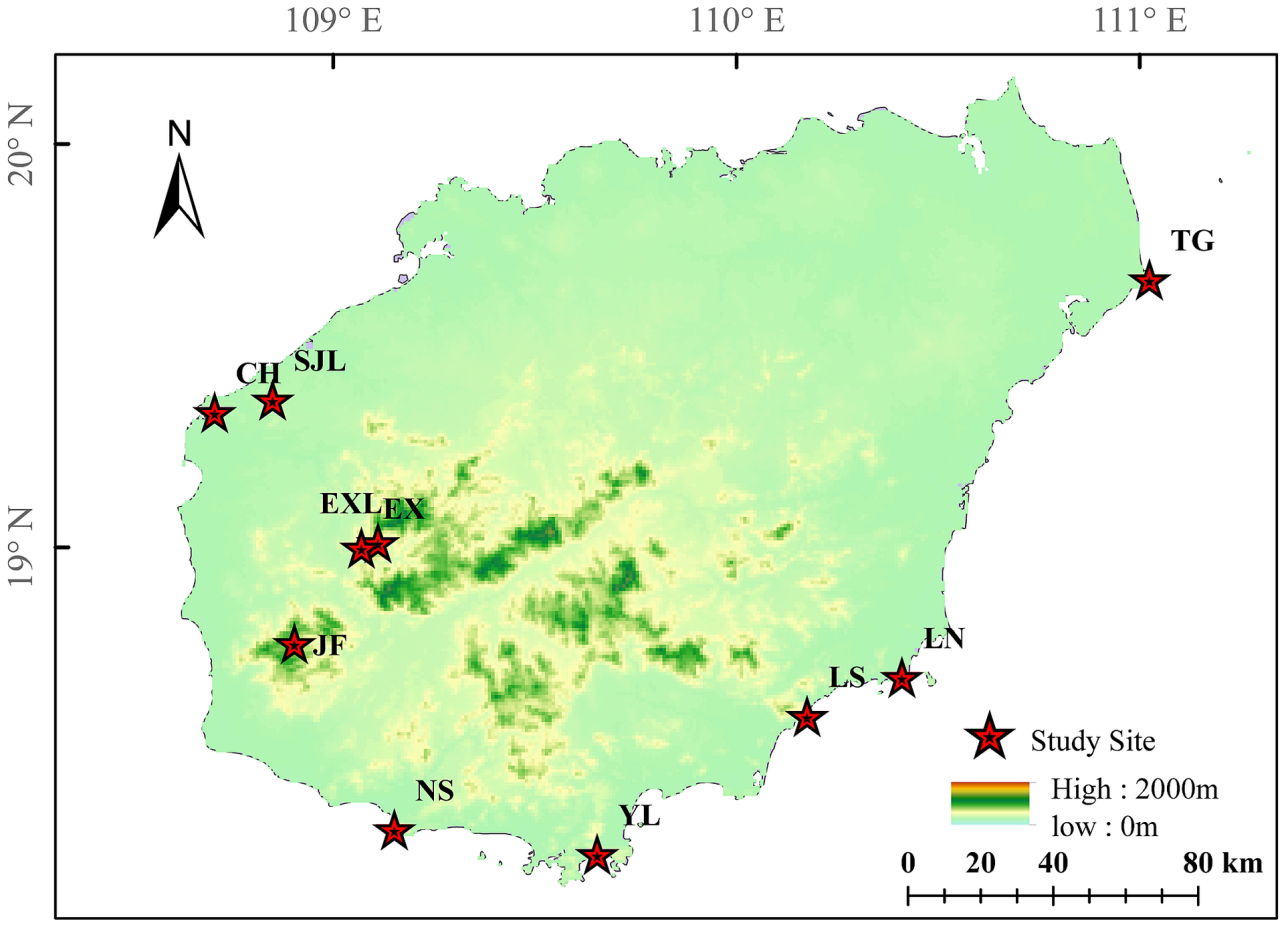
Locations of *D. cambodiana* populations. NS: Nanshan population in Sanya City; YL: Yalongwan population in Sanya City; LS: Boundary Island population in Lingshui County; LN: Lenanshan population in Wanning City; TG: TongGuling population in Wenchang City; CH: Changhualing population in Changjiang County; SJL: Sanjialing population in Changjiang County; JF: Jianfengling population in Ledong County; EXL: Exianling population in Dongfang City; EX: Nanlang population in Dongfang City.

Ten sampling sites of *D. cambodiana* on Hainan Island are shown in Figure 6. In total, 4,408,521 reads were detected. After low-quality chimeric sequences were filtered out, 770,998 high-quality sequences were obtained. In total, 7,513 OTUs were produced by clustering these sequences into OTUs with a 97% similarity.

**Figure 7:**
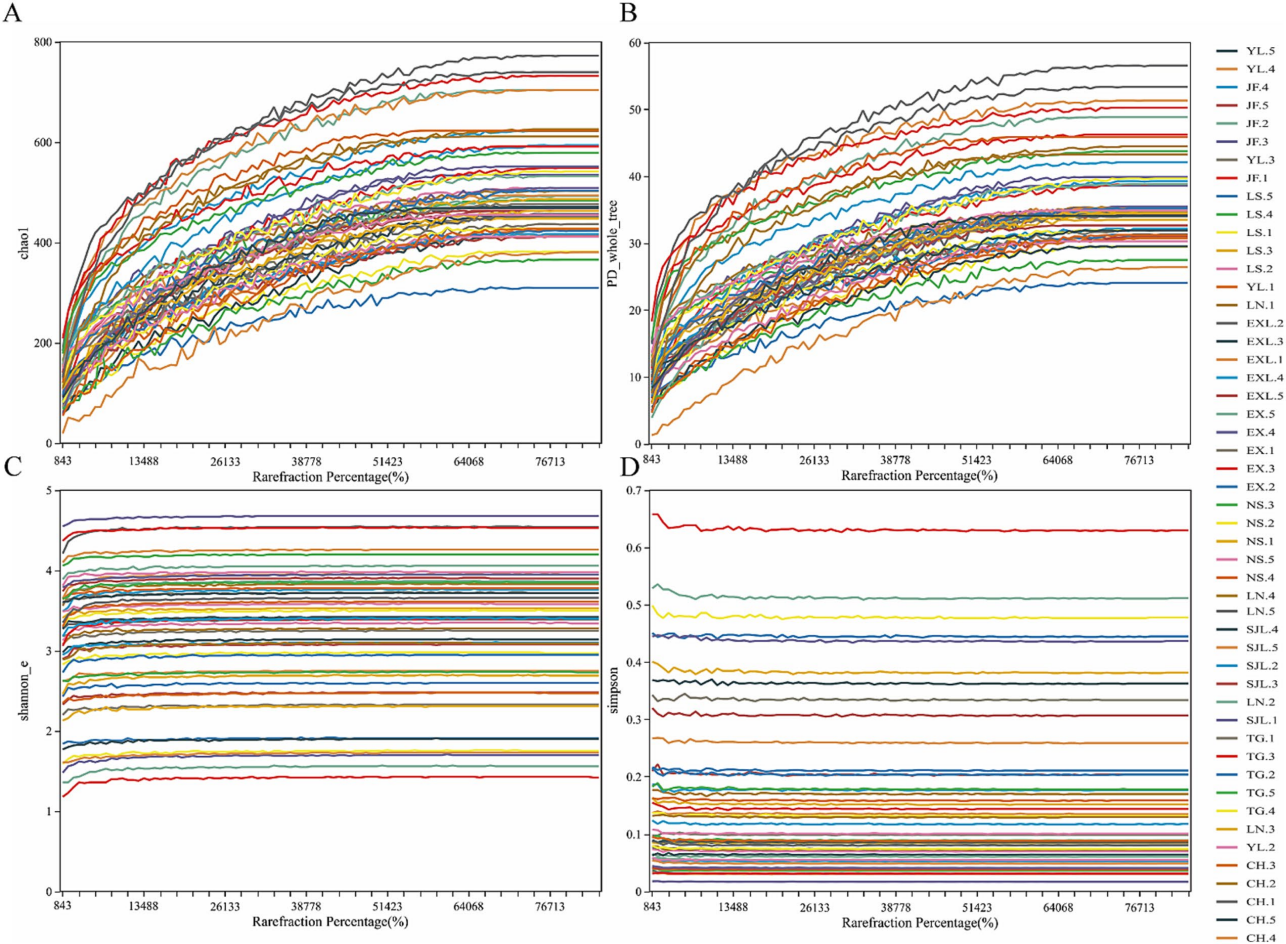
Rarefaction curves of endophytic fungal diversity indices in *D. cambodiana*. **(A)** Chao1 inde. **(B)** PD index. **(C)** Simpson inde. **(D)** Shannon index.

The rarefaction curve in Figure 7 was used to interpret species richness and evenness. The rarefaction curve showed that each sample group’s species richness and diversity indices (Chao1, PD, Simpson, and Shannon) gradually leveled off as the sequencing depth increased. These results showed that the sequencing depth and the number of biological replicates were adequate to represent the species richness and variety of the samples accurately.

**Figure 8:**
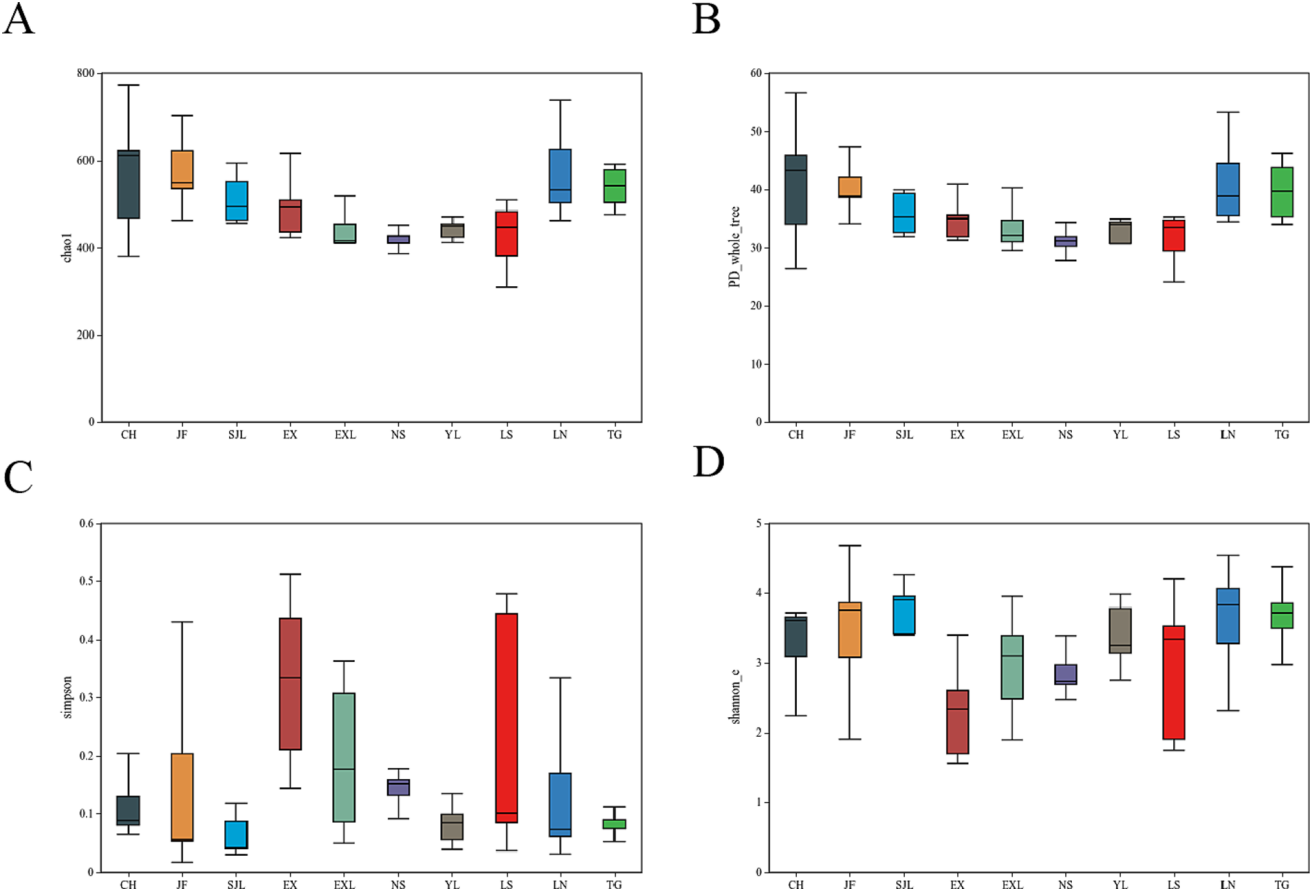
Boxplots of diversity indices for endophytic fungal communities in different populations of *D. cambodiana*. **(A)** Chao1 index. **(B)** PD index. **(C)** Simpson index. **(D)** Shannon index.

The Chao1 index (Figure 8A) indicated that the CH, JF, LN, and TG groups presented higher species richness, whereas the EX, NS, YL, and EXL groups presented lower species richness. However, no significant difference was found in species richness among the different groups (Wilcoxon test, *p* > 0.05). The CH group had the highest phylogenetic diversity, indicating that endophytic fungi in this group exhibited greater evolutionary divergence on the phylogenetic tree, with greater species diversity (Figure 8B). In contrast, the NS group had a lower PD index (Figure 8B), suggesting that the endophytic fungi in this group presented lower species diversity. The CH, SJL, and JF groups exhibited elevated Shannon indices (Figure 8C), signifying higher species diversity and more equitable distribution, while the EX group demonstrated reduced species diversity (Figure 8D). However, there were no statistically significant variations in the distribution of dominating species or species diversity among endophytic fungal communities in the several regions where *D. cambodiana* was found (Wilcoxon test, *p* > 0.05). The findings indicated that the diversity and structure of endophytic fungal communities were largely consistent throughout the study area, demonstrating no notable geographic variations. The findings suggest that geographical location has a lesser effect on the structure of *D. cambodiana* endophytic communities than tissue specificity.

**Figure 9:**
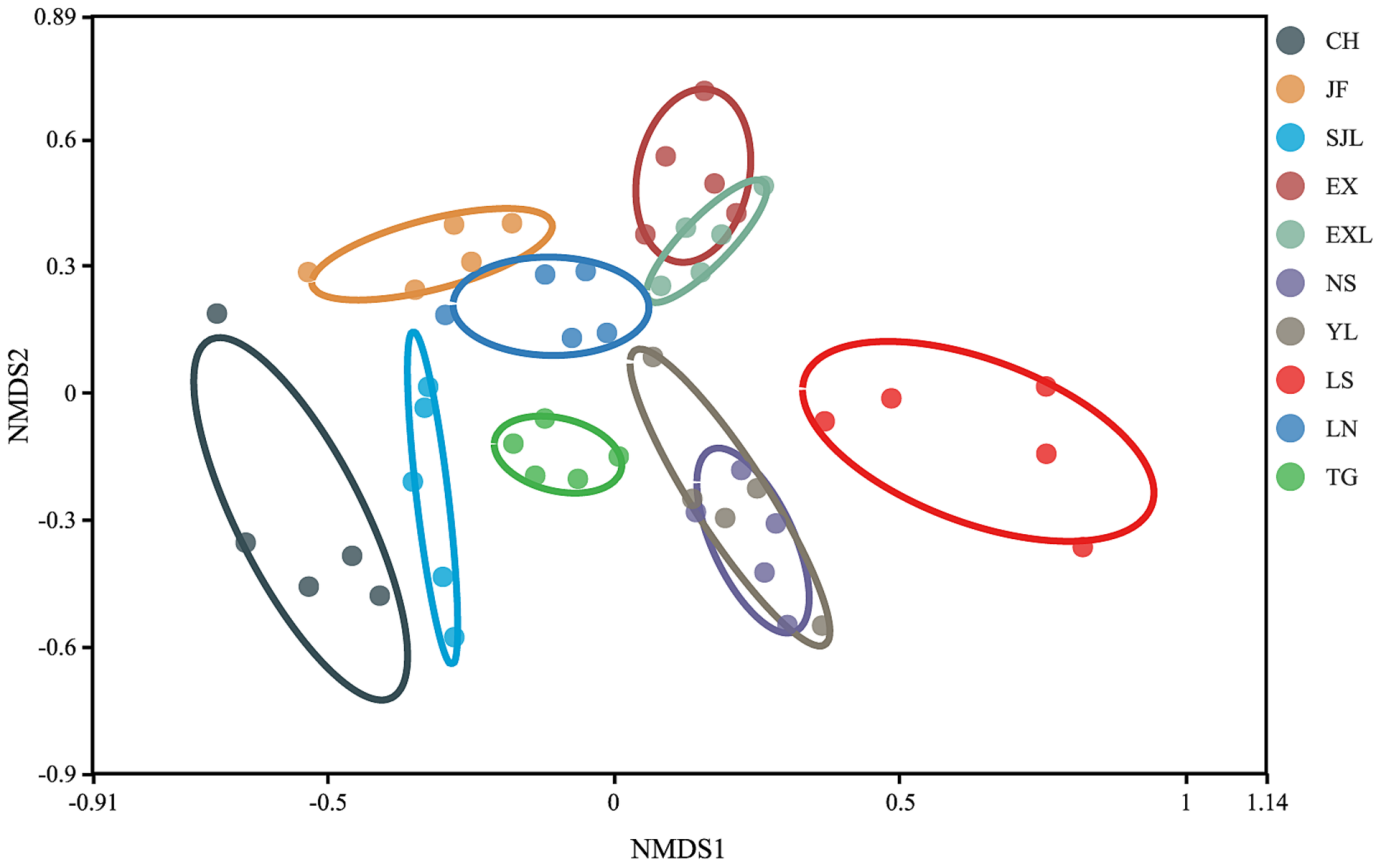
Non-metric multidimensional scaling (NMDS) of endophytic fungal communities in different populations of *D. cambodiana*.

The NMDS plot (Figure 9) showed the distribution of samples from each group along two primary coordinate axes. CH and LS showed greater variation in community structure within these groups. In contrast, samples from the TG and EXL groups were more tightly clustered, suggesting a more similar community structure within these groups. The degree of separation between different sample groups also varied. Samples from the same region generally clustered together and maintained a certain distance from other areas, indicating differences in community structure between groups. Overall, endophytic communities in the stems of *D. cambodiana* in geographically proximate regions exhibited greater similarity. For example, locations along the northwest coast, such as CH and SJL, inland plains, such as EXL and EX, and the southern coast, including NS and YL, had similar community structures. However, this pattern was not strictly consistent across all areas. For example, TG, the most geographically distant group, did not show the greatest divergence in its endophytic community. In contrast, the endophytic communities in southeastern areas, such as JF, LS, and LN, exhibited relatively significant differences. This indicates that, in addition to physical distance, factors like altitude and specific climatic variables may also affect the composition of endophytic communities in the stems of *D. cambodiana*.

**Figure 10:**
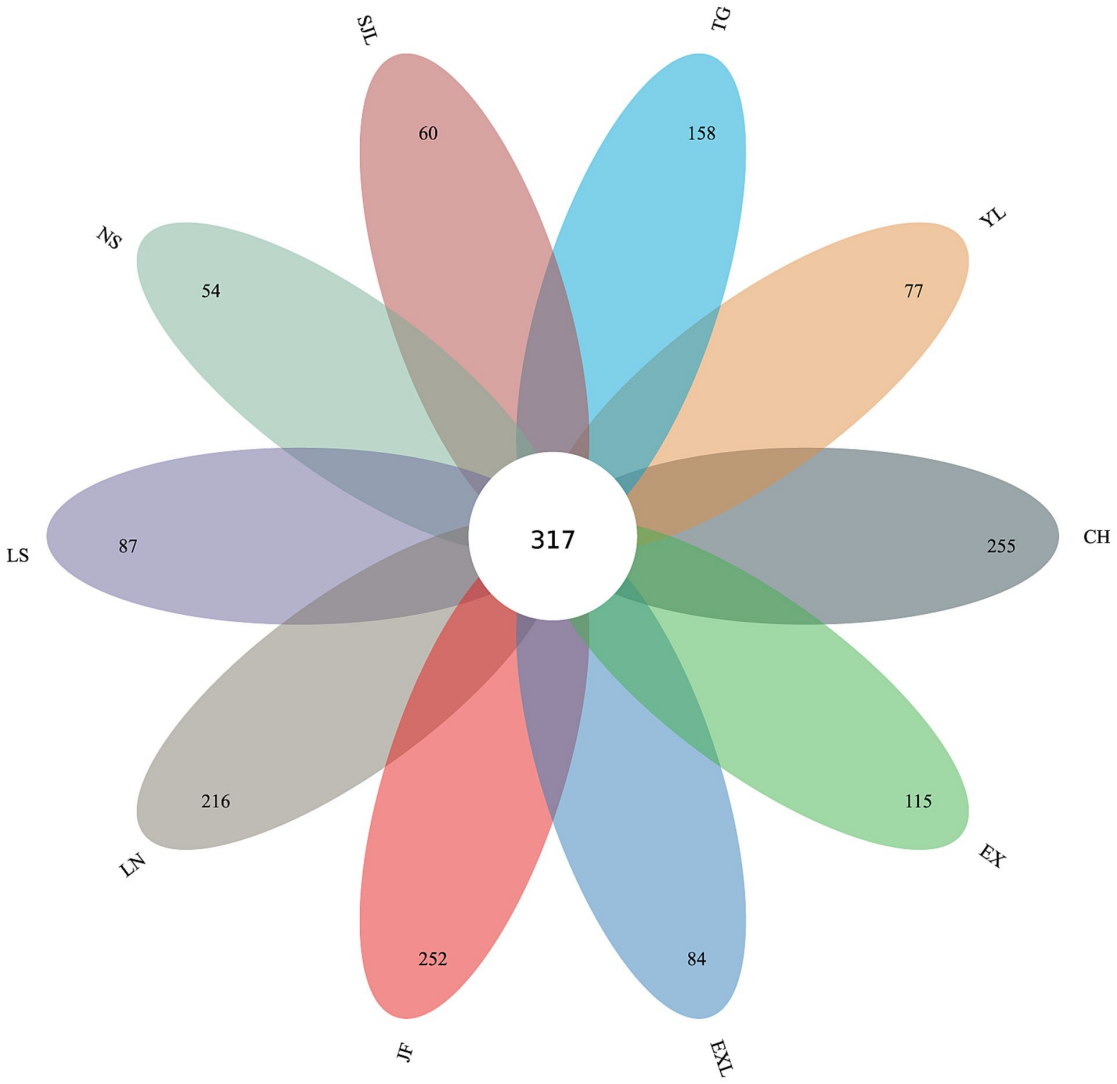
Petal diagram of shared and unique OTUs of endophytic fungi across different sample groups.

The petal diagram (Figure 10) shows that the unique OTU counts for groups JF, LN, LS, NS, YL, EX, EXL, CH, SJL, and TG are 252, 216, 87, 54, 77, 115, 84, 255, 60, and 158, respectively, with 317 core OTUs shared across all groups. The shared and unique OTUs of endophytic fungi at the genus and species taxonomic levels across different regions are also illustrated. The diagram shows 317 shared OTUs across all groups, indicating a core endophytic fungal community. In contrast, each region had unique OTUs ranging from 54–255, suggesting a distinct composition of endophytic fungal communities in each region. The number of unique OTUs reflects each region’s diversity and uniqueness of endophytic fungal communities. Regions with a higher diversity of unique OTUs (e.g., CH, EX, JF, and LN) show greater species uniqueness, suggesting that geographic location may significantly influence the composition of endophytic fungal communities.

**Figure 11:**
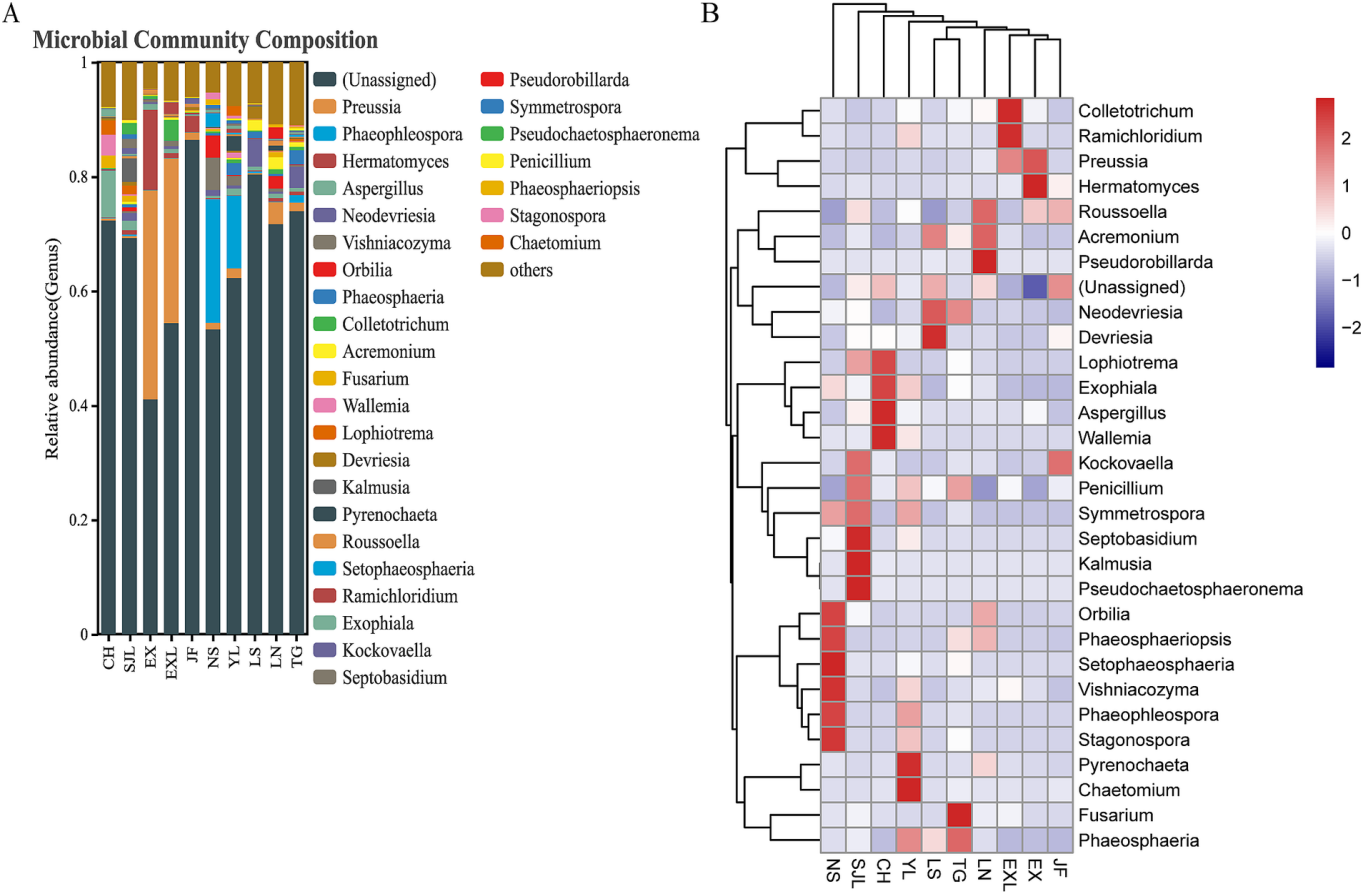
Relative abundance and heat map of endophytic fungal genera across different populations of *D. cambodiana*. **(A)** The stacked bar plot of relative abundance of fungal genera in each sample group. **(B)** The heat map of relative abundance of fungal genera.

Relative abundance and heat map of endophytic fungal genera across different populations of *D. cambodiana* (Figure 11) shows that Ascomycota was the dominant fungal phylum in all groups (Figure 11A), with a relative abundance ranging from 93.91 to 67.05%. The main classes within Ascomycota were Dothideomycetes, Sordariomycetes, and Eurotiomycetes, while the primary orders across groups were Pleosporales, Capnodiales, Chaetothyriales, and Hypocreales (Figure 11A). In the NS group, the dominant genera included *Orbilia* (3.97%), *Setophaeosphaeria* (2.49%), *Vishniacozyma* (3.79%), *Phaeophleospora* (21.62%), and *Stagonospora* (1.24%). The SJL group was dominated by *Septobasidium* (1.59%), *Kalmusia* (3.93%), and *Pseudochaetosphaeronema* (1.93%). In the CH group, the main dominant genera were *Aspergillus* (5.13%) and *Wallemia* (3.95%), whereas *Pyrenochaeta* (2.59%) and *Chaetomium* (1.65%) were the dominant genera in the YL group. In the LS group, *Devriesia* (2.15%) was dominant; in the TG group, *Fusarium* (13.94%) was dominant; in the LN group, *Pseudorobillarda* (1.96%) was dominant; in the EXL group, *Colletotrichum* (3.66%) and *Ramichloridium* (1.98%) were dominant; in the EX group, *Hermatomyces* (14.26%) was dominant; and in the JF group, no genera had an abundance greater than 1%. Some geographically close regions shared dominant bacterial genera within their endophytic communities. For example, *Preussia* in EXL and EX, *Phaeophleospora* and *Stagonospora* in YL and NS.

#### 3.2.1 Metagenomic sequencing, assembly, and annotation

Samples from healthy, short-term, and long-term post-injury *D. cambodiana* were collected, with three replicates per treatment, for metagenomic sequencing of endophytic fungi. In total, 113,928,065 raw reads were obtained from this metagenomic sequencing analysis. After removing low-quality sequences, adapters, and chimeric reads, 111,639,185 high-quality clean reads were retained. These reads had high Q20 and Q30 scores, ensuring reliable data. During the assembly, these clean reads were further assembled into 3,413,555 scaftigs.

#### 3.2.2 Functional enrichment of endophytic fungal communities during resin formation

**Figure 12:**
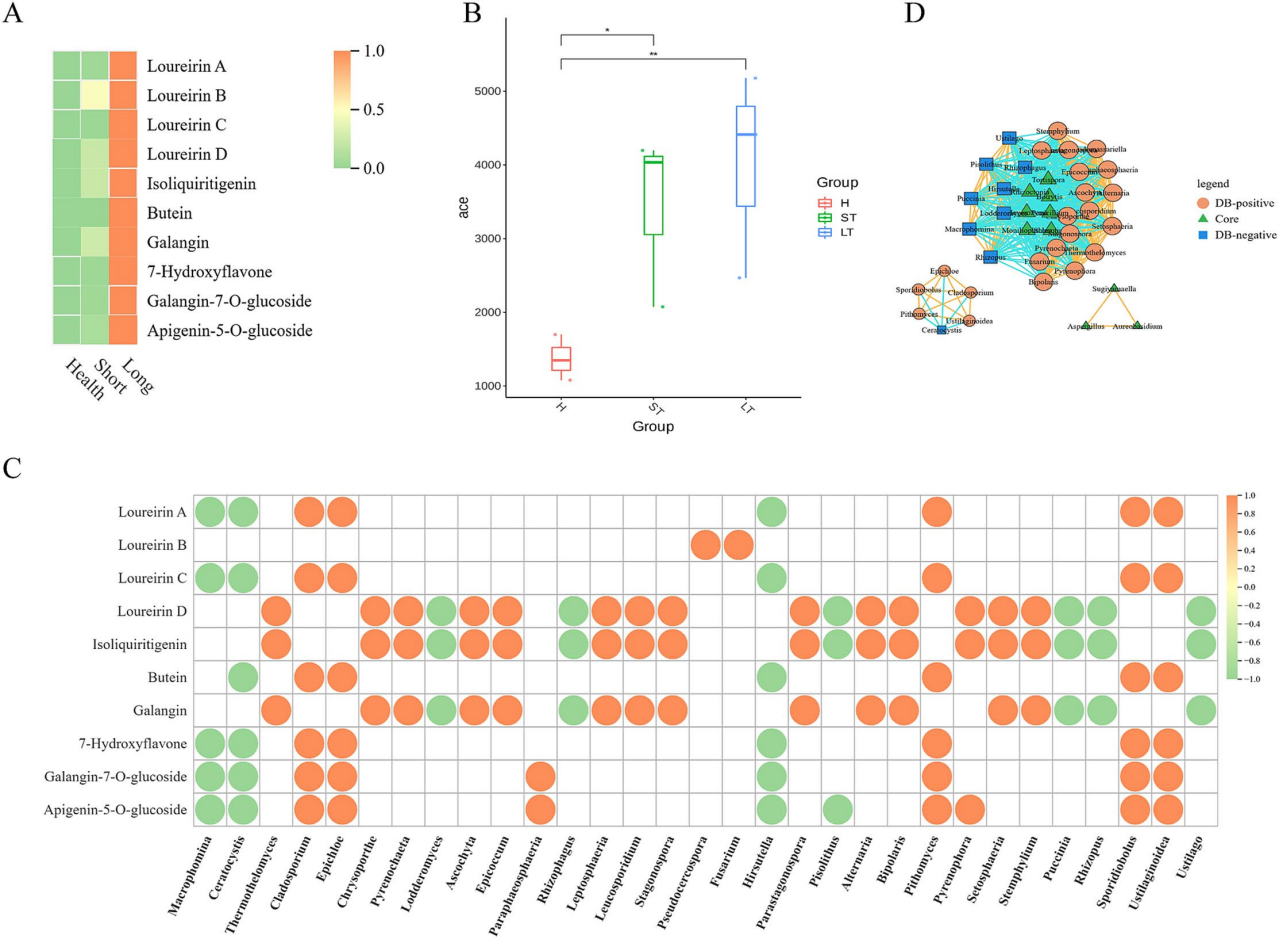
Correlation between metabolites and microbiota in *D. Cambodian* across different injury time periods. **(A)** Heatmap of Representative compounds of DB in different period after wounding. **(B)** Alpha-diversity of fungal community in different period after wounding. **(C)** Dot diagram of Correlation analysis between DB compounds and fungal genus. **(D)** Correlation network diagram among core and Differential community during different period after wounding.

We further explored the functional composition and diversity of endophytic fungal communities during the resin formation process. Over time after injury, the content of representative chemical components in *D. cambodiana* significantly increased (Figure 12A), indicating the formation and accumulation of dragon’s blood under wound stress. During this process, the taxonomic diversity of the endophytic fungal communities progressively increased as well as the number of genes (Figure 12B). Correlation analysis indicates that certain fungal genera (e.g., *Cladosporium*, *Epichloe*, *Pithomyces*, *Sporidiobolus*, and *Fusarium*) are positively correlated with dragon’s blood components, possibly promoting resin production, whereas other genera (e.g., *Macrophomina*, *Ceratocystis*, *Hirsutella*, and *Rhizopus*) are negatively correlated (Figure 12C). Correlation network between the dominant and significantly changed communities shows that during the resin formation process, most core communities show a negative correlation with both resin formation positively correlated and negatively correlated communities. Additionally, some core communities do not exhibit any correlation with resin-related communities, suggesting that the core communities may primarily function to maintain the microenvironmental homeostasis of *Dracaena cambodiana* rather than directly contributing to resin production. Meanwhile, the resin formation positively correlated and negatively correlated communities exhibit a significant negative correlation, indicating an “antagonistic” interaction between these groups (Figure 12D).

**Figure 13:**
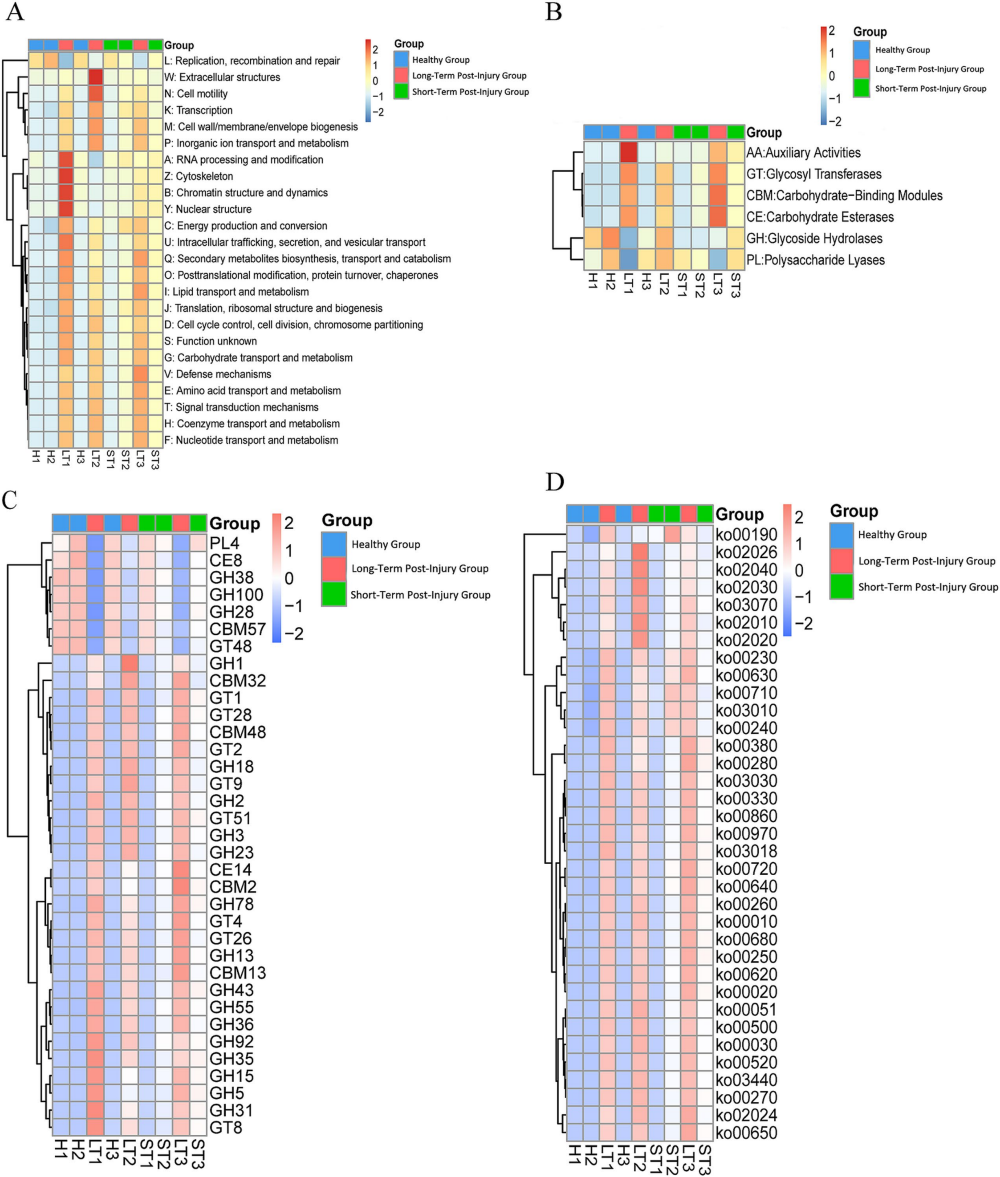
Functional profiling of endophytic unigenes across different periods post damage. **(A)** A heat map of unigene functional classifications based on eggNOG database categories. **(B)** A heat map of carbohydrate-active enzyme (CAZy) functional families. **(C)** A detailed heat map of the glycosyl transferase (GT) family. **(D)** A heat map of enriched KEGG pathways. Warmer colors (red) represent greater functional abundance, and cooler colors (blue) indicate lower abundance. G1: healthy, G2: short-term postinjury, and G3: long-term postinjury.

The functions of endophytes significantly differed among the different periods. The eggNOG database shows that the number of unigenes linked to various terms, such as defense mechanisms, signal transduction mechanisms, transport and catabolism, resin transport and metabolism, amino acid transport and metabolism, extracellular structure, secondary metabolite biosynthesis, etc., increased from healthy samples to samples that had sustained long-term damage (Figure 13A). According to the CAZy database, the number of unigenes associated with glycoside hydrolases consistently decreased, whereas the number of unigenes related to auxiliary activities, carbohydrate esterases, and glycosyl transferases continuously increased (Figure 13B). According to a different study, most flavonoids generated over time during resin production were glycosylated (Liu et al., 2022). In this study, many unigenes annotated as glycosyl transferase families, including GT28, GT51, GT4, GT1, GT9, GT2, and GT26, increased throughout the resin formation process (Figure 13C). Therefore, unigenes associated with various KEGG pathways, such as carbon fixation, propanoate metabolism, pyruvate metabolism, degradation of valine, leucine, and isoleucine, metabolism of alanine, aspartate, and glutamate, RNA degradation, aminoacyl-tRNA biosynthesis, the citrate cycle (TCA cycle), methane metabolism, ABC transporters, quorum sensing, two-component systems, flagellar assembly, metabolism of amino sugars and nucleotide sugars, arginine and proline metabolism, and porphyrin metabolism, were observed to increase (Figure 13D).

Next, we focused on the unigenes annotated in the defensive responses and resin formation pathways. In the annotation of plant-pathogen interactions, there was a gradient increase from healthy to long-term damaged samples. Moreover, in the annotation of apoptosis pathways, the annotation quantity decreased from healthy samples to short-term damaged samples but significantly increased in long-term damaged samples. Genes associated with phenylalanine metabolism, starch, and sucrose metabolism, and isoflavone biosynthesis were identified in short-term and long-term damaged samples but not in the healthy samples. The activity of the flavonoid biosynthesis-related pathways increased in healthy samples, decreased in short-term damaged samples, and partially recovered in long-term wounded samples.

## 4 Discussion

### 4.1 The main factors influencing the community structure of endophytic fungi in *D. cambodiana*

The effect of tissue specificity was greater than that of regional specificity. In different tissues of *D. cambodiana*, the endophytic fungal communities in the bark, cortex, and secondary growth regions exhibited some degree of overlap, and each tissue also displayed unique dominant genera. Specifically, the bark had unique dominant genera, such as *Lophiotrema* and *Lophiostoma*, whereas the cortex and secondary growth region contained *Neournula* and *Pseudallescheria* as the dominant genera, respectively. In the primary growth region, *Graphiola*, *Symmetrospora*, *Phaeosphaeriopsis*, and *Mycosphaerella* were the main dominant genera, indicating that different tissues possess distinct endophytic fungal community characteristics, probably related to their ecological functions and biological roles. As a monocotyledonous plant, *D. cambodiana* is tree-like due to its secondary phloem. The stem can be divided into loosely structured primary and densely packed, harder secondary tissue (Jura-Morawiec, 2017; Zhang et al., 2023). The findings demonstrate that primary and secondary tissues play distinct roles in the normal growth and development of *D. cambodiana*. Primary tissue is more fragile and susceptible to decay and death upon damage. Dead primary tissue has a negligible effect on the overall survival of trees, leading older trees to often become hollow.

Further subdividing stem tissues, we found that the community structure varied among tissues. These divergent communities may be evolutionarily related to the unique stem structure of *D. cambodiana*, warranting further investigation. The interaction between plants and endophytic fungi is a result of long-term coevolution. Several studies have suggested that plant endophytic fungal communities can be vertically inherited through sexual reproduction, thus retaining this symbiotic relationship (Hodgson et al., 2014; Shahzad et al., 2018). In *D. cambodiana*, the genera *Cladophialophora* and *Entoloma* are abundant in both roots and seeds, suggesting that these communities may also be transmitted through seeds. These communities may contribute significantly to the growth and development of *D. cambodiana*.

Compared to tissue specificity, regional specificity differences were smaller, and we found that these differences may be related to the local climate and geographical location. For example, the CH region in the northwest is relatively arid and has the highest species and phylogenetic diversity. In contrast, the TG region is near the coast and is influenced by a maritime climate with high humidity and temperature, making *Fusarium* the dominant genus.

### 4.2 Endophytic fungi might play multiple roles in the resin formation process of *D. cambodiana*

The formation of dragon’s blood resin is a complex plant defense response involving multiple mechanisms, such as wound recognition, signal transduction, apoptosis, and the regulation of secondary metabolites such as flavonoids, as well as the transport and modification of flavonoids (Zhu et al., 2016; Ding et al., 2018). Hydrogen peroxide is a significant signaling molecule in the resin formation process of *D. cambodiana* (Liu et al., 2022). In response to damage, *D. cambodiana* activates and upregulates various metabolic pathways across different stages, including sucrose and resin metabolism in the early stages, flavonoid biosynthesis in the middle to late stages, and flavonoid glycosylation in the final stages. This complex process ultimately leads to resin formation, preventing further damage.

Significant changes were found in the gene abundance of endophytic fungi at different stages postinjury. This finding suggested that these fungi may be involved in multiple aspects of the abovementioned processes. For example, postinjury annotations revealed several unigenes involved in resin metabolism, sucrose metabolism pathways, signal transduction, phenylalanine metabolism, and isoflavone synthesis pathways. These genes were absent in the endophytic fungal communities of healthy *D. cambodiana*. Additionally, multiple glycosylation gene families were found in the postinjury community. Previous reports have indicated that flavonoid glycosylation is an essential process in the later stages of resin formation (Liu et al., 2022), facilitating the transport of flavonoids from synthesis sites to the wound site and reducing autotoxic effects on the plant. Our findings suggested that endophytic fungi may be crucial in this process.

Additionally, our study observed that certain endophytic fungi, including *Cladosporium*, *Fusarium*, and *Trichoderma* species, may play a role in promoting resin production and accumulation in *D. cambodiana*, which aligns with their known roles in other plants. *Cladosporium* species promote plant adaptation and health by producing secondary metabolites, enhancing plant defense responses. Experimental results indicate that *Cladosporium T2* isolate significantly stimulates tomato seedling growth through the secretion of volatile compounds and hydrolytic enzymes (Răut et al., 2021). *Fusarium* species, particularly non-pathogenic strains, have been shown to secrete secondary metabolites like volatile organic compounds (VOCs) that help suppress pathogenic fungi and nematodes, thereby improving plant health and growth (Al-Ani, 2019). These fungi may not only contribute to resin accumulation but also support the overall health and defense mechanisms of *D. cambodiana*, facilitating the resin formation process.

Furthermore, changes in the endophytic fungal community during resin formation did not significantly indicate pathogenic development. Several common highly pathogenic plant fungi were not notably enriched, suggesting that the primary fungi that induce resin formation in *Dracaena* may not be directly related to pathogenicity.

### 4.3 Considerations for selection of strains that effectively induce resin formation

Although several research groups have used fungi to induce resin production in *D. cambodiana* (Gong and Guo, 2009; Ding et al., 2020), the production efficiency has been relatively low, and many researchers have used highly pathogenic fungi as elicitors, raising safety concerns. However, no related technology is widely used in practical production. This study presents the following recommendations for the development of a fungus-based induction method:

1. Candidate fungi were isolated and extracted based on the subdivided stem tissue structures. The results indicated apparent differences in endophytic fungal communities across different stem tissues, suggesting that fungal strains isolated from these tissues also vary significantly. Considering the different resin-producing capacities of these tissues, secondary stem tissues of *Dracaena* should be carefully selected as sources of candidate fungi.
2. The time after damage should be considered when selecting and isolating candidate fungi from resin-producing regions. We found significant changes in the gene function abundance of *D. cambodiana* endophytes at different postinjury stages. Therefore, isolating candidate strains with specific functions should be more accessible during resin production, potentially improving resin yield.
3. When selecting suitable strains as resin inducers, although our study did not directly compare the effectiveness of different strains in resin induction, we observed significant differences in fungal community structure across different plant tissues through the analysis of endophytic fungal communities. For instance, *Fusarium* species dominate the roots, while a more complex and diverse community structure is observed in the leaves and stems. Specifically, the endophytic communities in the leaves and stems exhibit higher species diversity and richness (as shown in Figure 5). These findings suggest that the selection of strains should not only be based on their species but also consider their parasitic characteristics in specific plant tissues. For example, although a single strain can induce resin production in certain tissues, its effects may vary depending on the tissue characteristics and the strain’s parasitic capabilities. Therefore, selecting dominant strains from specific tissues for resin induction can enhance resin yield while minimizing negative impacts on the overall health of the plant.

The findings of this study revealed that several novel genes linked to pathways implicated in dragon’s blood resin synthesis emerged in the stem community following damage. These gene expressions were unlikely to be solely due to changes in the abundance of individual species. Future studies should focus on fungal isolation and inoculum development. These strategies could enhance resin induction efficiency but were beyond the scope of this study. Future work could explore the feasibility of isolating specific fungal strains from different tissues and developing optimized inoculum preparations, which might be critical for improving the scalability and practicality of resin induction in *D. cambodiana*.

## Data Availability

The datasets generated or analyzed during this study are available in the National Center for Biotechnology Information (NCBI) repository, https://www.ncbi.nlm.nih.gov/, accession numbers: PRJNA1185629, PRJNA1187799, PRJNA1185199, and PRJNA1185465.

## Footnotes

^1^ http://www.iucnredlist.org
